# A survey of retail prices of antimicrobial products used in small-scale chicken farms in the Mekong Delta of Vietnam

**DOI:** 10.1186/s12992-019-0539-x

**Published:** 2020-01-14

**Authors:** Nguyen T. T. Dung, Bao D. Truong, Nguyen V. Cuong, Nguyen T. B. Van, Doan H. Phu, Bach T. Kiet, Chalalai Rueanghiran, Vo B. Hien, Guy Thwaites, Jonathan Rushton, Juan Carrique-Mas

**Affiliations:** 1 Wellcome Vietnam Africa Asia Program, Oxford University Clinical Research Unit, 764, Vo Van Kiet, District 5, Ho Chi Minh City, Vietnam; 2 InterRisk program, Faculty of Veterinary Medicine, Kasetsart University, Bangkok, Thailand; 3 Faculty of Animal Science and Veterinary Medicine, University of Agriculture and Forestry, Ho Chi Minh City, Vietnam; 4 Sub Department of Animal Health and Production, Cao Lanh, Vietnam; 5 Department of Veterinary Public Health, Faculty of Veterinary Medicine, Kasetsart University, Bangkok, Thailand; 6 Institute of Infection and Global Health, University of Liverpool, Liverpool, UK; 7 Centre for Tropical Medicine and Global Health, Nuffield Department of Medicine, Oxford University, Oxford, UK

**Keywords:** Mekong Delta, Vietnam, Chicken, Poultry, Antimicrobial, Animal daily dose, Cost

## Abstract

**Background:**

In the Mekong Delta region of Vietnam, high quantities of products containing antimicrobial are used as prophylactic and curative treatments in small-scale chicken flocks. A large number of these contain antimicrobial active ingredients (AAIs) considered of ‘critical importance’ for human medicine according to the World Health Organization (WHO). However, little is known about the retail prices of these products and variables associated with the expense on antimicrobials at farm level. Therefore, the aims of the study were: (1) to investigate the retail price of antimicrobials with regards to WHO importance criteria; and (2) to quantify the antimicrobial expense incurred in raising chicken flocks. We investigated 102 randomly-selected small-scale farms raising meat chickens (100–2000 per flock cycle) in two districts in Dong Thap (Mekong Delta) over 203 flock production cycles raised in these farms. Farmers were asked to record the retail prices and amounts of antimicrobial used.

**Results:**

A total of 214 different antimicrobial-containing products were identified. These contained 37 different AAIs belonging to 13 classes. Over half (60.3%) products contained 1 highest priority, critically important AAI, and 38.8% 1 high priority, critically important AAI. The average (farm-adjusted) retail price of a daily dose administered to a 1 kg bird across products was 0.40 cents of 1 US$ (₵) (SE ± 0.05). The most expensive products were those that included at least one high priority, critically important AAI, as well as those purchased in one of the two study districts. Farmers spent on average of ₵3.91 (SE ± 0.01) on antimicrobials per bird over the production cycle. The expense on antimicrobials in weeks with disease and low mortality was greater than on weeks with disease and high mortality, suggesting that antimicrobial use had a beneficial impact on disease outcomes (*χ*^2^ = 3.8; *p* = 0.052). Farmers generally used more expensive antimicrobials on older flocks.

**Conclusions and recommendation:**

The retail prices of antimicrobial products used in chicken production in Mekong Delta small-scale chicken farms are very low, and not related to their relevance for human medicine. Farmers, however, demonstrated a degree of sensitivity to prices of antimicrobial products. Therefore, revising pricing policies of antimicrobial products remains a potential option to curb the use of antimicrobials of critical importance in animal production.

## Background

Antimicrobial resistance (AMR) is a global health concern and excessive antimicrobial use (AMU) in animal production is one of the contributing factors [1]. The AMR situation has reached critical levels, and countries are being urged to take immediate action to mitigate the problem [2]. The practice of purchasing antimicrobials ‘over the counter’ without a prescription is widespread in many low- and middle-income countries (LMICs) [3]. This is also common practice in Vietnam, a country that currently ranks as the 15th most populous in the world (~ 97 M in 2019), in spite of existing legislation restricting access to antimicrobials for human use without prescription [4]. In contrast, antimicrobials intended for animal use can be legally purchased without a prescription by anyone from any of the approximately 10,000 veterinary drug shops across the country [5].

In 2011, the World Health Organization of the United Nations (WHO) ranked antimicrobial active ingredients (AAI) based on prioritization criteria for human medicine. This list has been modified on several occasions, and in 2018 the highest priority, critically important AAI category included 3rd and 4th generation cephalosporins, glycopeptides, macrolides, ketolides, polymyxins and quinolones [6].

There is growing consensus that use of antimicrobials of critical importance for human medicine in animals should be restricted/reduced [7–9]. However, a large number of AAIs considered by WHO to be of critical importance are currently used in animal production worldwide [10]. The Mekong Delta of Vietnam is regarded as a hotspot for AMU in animal production [11–14], and levels of use of AAIs considered of critical importance are high. A recent study on small-scale chicken farms in the Mekong Delta indicated that 76.2% antimicrobial products contained AAIs of critical importance according to WHO [14]. It has been suggested that antimicrobials used in animal production in Vietnam are very affordable. A study on the 10 most popular products used by farmers showed that the average cost of a daily dose was 0.56 cents of 1 US$ (range ranged from 0.19 to 1.03) [15]. It is not clear whether retail prices reflect their AAIs composition and their relevance to human health, and to what extent low pricing contributes to excessive AMU in animal production in Vietnam. We investigated antimicrobial products used in a sample of 112 randomly selected small-scale commercial farms (203 flocks) raising native chickens in the Mekong Delta of Vietnam. We quantified AAIs contained in these products as well as their retail prices. The aims of this study were: (1) to investigate the price of antimicrobials with regards to WHO importance criteria and formulation (single AAIs or combined AAIs) and (2) to investigate changes in the expense on antimicrobials over the production cycle.

## Results

### Total and weekly expense on antimicrobials

**Fig. 1:**
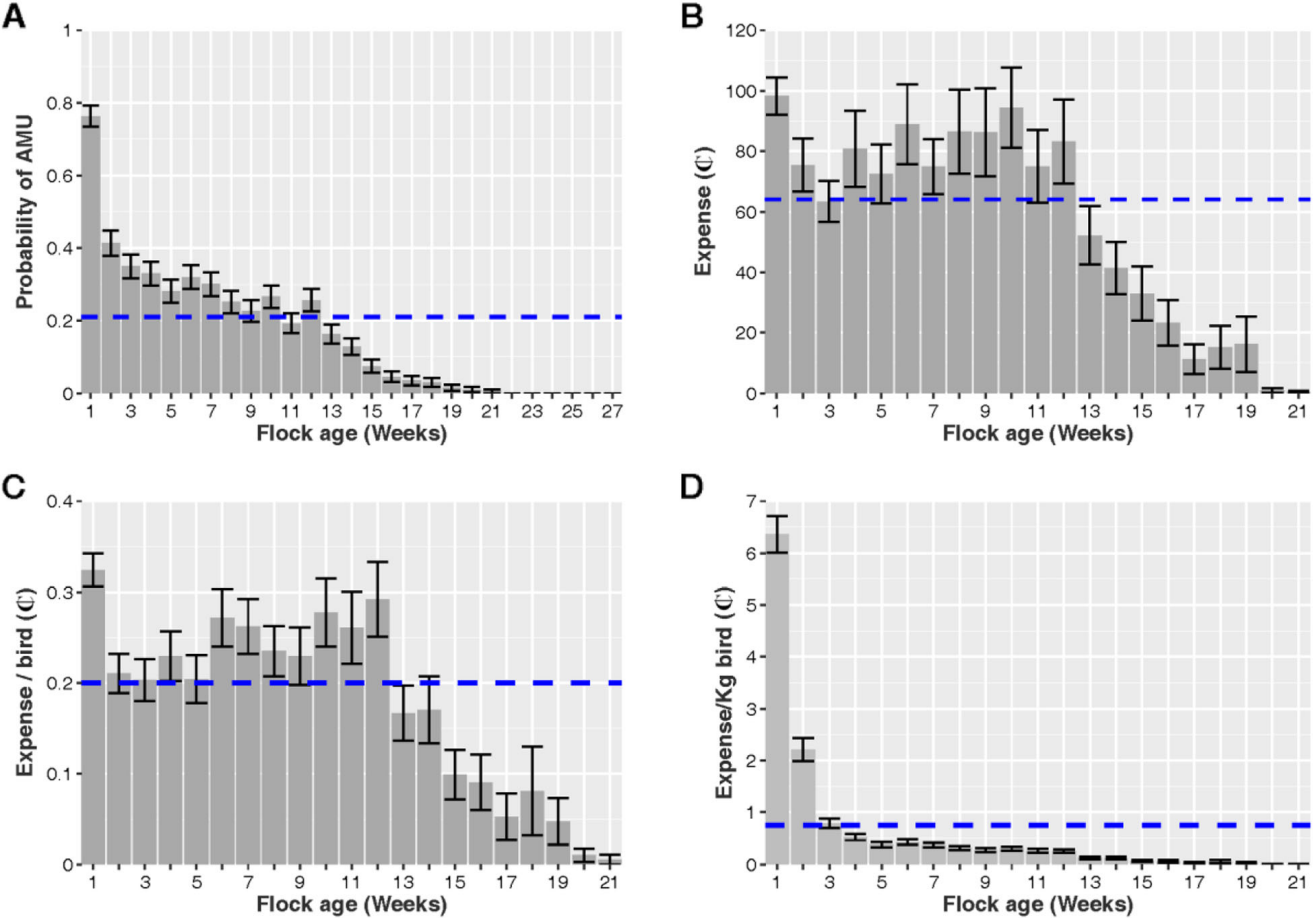
**a** Probability of flocks using antimicrobials by age (weeks); **b** Weekly expense (per flock) on antimicrobials during the flock production cycle by age; **c** Total expense (per bird) on antimicrobials; **d** Total expense per kilogram of bird. Costs are expressed in cents of 1 US$ (₵). The blue lines represent the crude (unadjusted) mean across all observation weeks. Vertical bars represent the standard error of the mean (SE)

Data on AMU and their retail prices were obtained from 203 complete cycles of native chicken flocks raised for meat in 102 farms. The median flock size at restocking was 300 [Inter-quartile range (IQR) 200–495], and the median duration of production cycles was 18 [IQR 16–20] weeks. The median cumulative mortality over the whole production cycle across flocks was 14.10% [IQR 6.8–29.2]. The average probability of AMU by week across flock cycles was 0.21 (SE ± 0.02) (Fig. 1a). The total expense on antimicrobials by farmers over the 203 cycles of production was US$2529.50 (Fig. 1b). The average expense on antimicrobials per flock cycle was US$12.50. The average cumulative expense on antimicrobials to raise one bird was ₵3.91 (SE ± 0.03). On average, farmers spent ₵64.07 (SE ± 2.45) on antimicrobials per week (Fig. 1b), and ₵0.20 (SE ± 0.01) per bird per week (Fig. 1c).

The highest probability of AMU corresponded to the first week of age of flocks (0.76; SE ± 0.03), decreasing thereafter (Fig. 1a). The weekly total expense on antimicrobials was highest during the 8–12 period week, peaking on week 10 (per flock mean ₵128.60; SE ±13.36) (Fig. 1b). After week 13, overall expense on antimicrobials decreased considerably (≤₵52.19 per week). In relation to live chicken weight, the weekly average expense on antimicrobials was was ₵0.75 per kg of live bird (SE ± 0.05). The highest expense corresponded to the first week of production (per flock mean ₵6.36; SE ±0.35) and quickly decreasing thereafter (≤₵2.21 per week) (Fig. 1d).

### Antimicrobials and disease

**Fig. 2:**
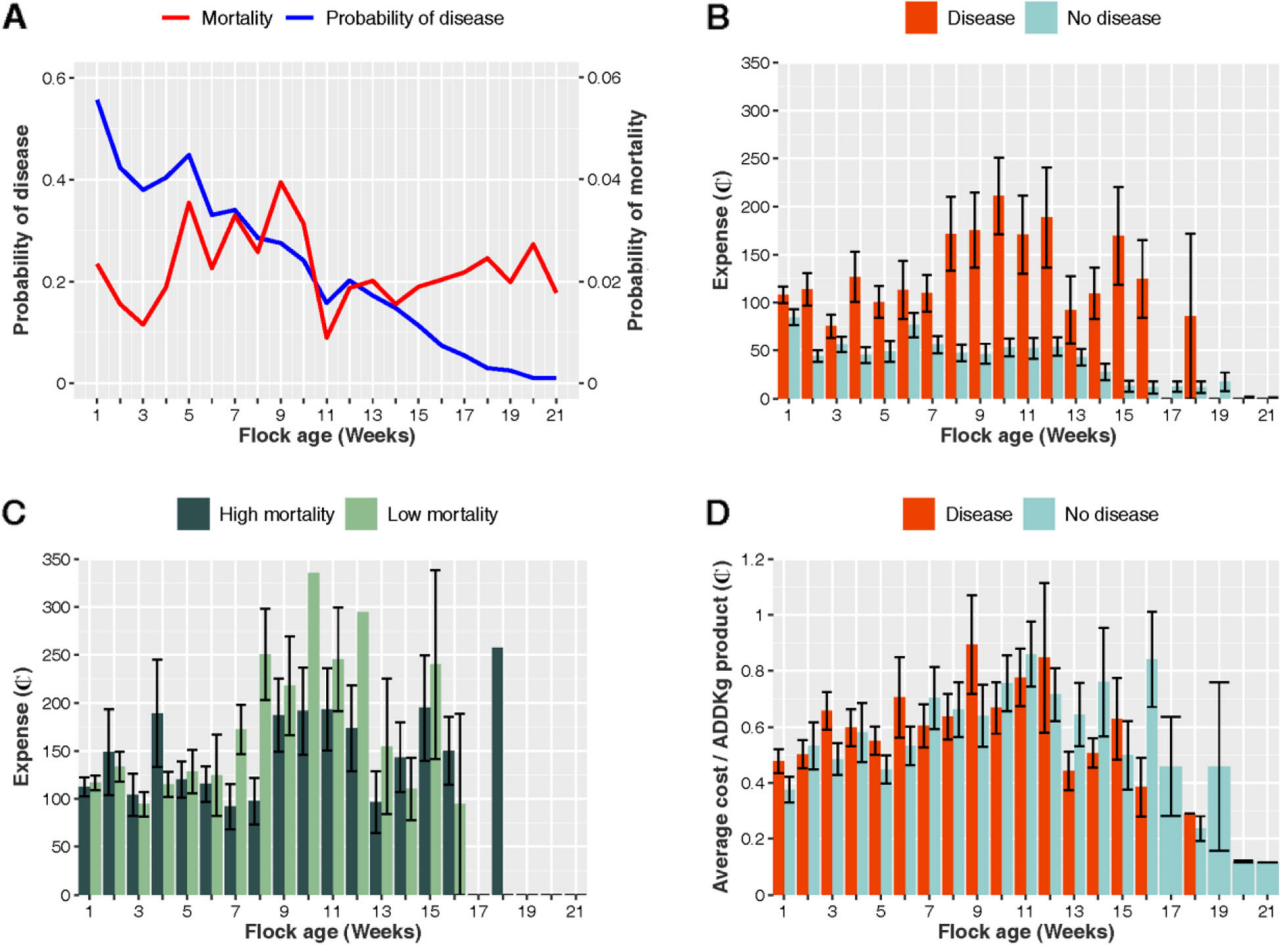
**a** Probability of disease across all flocks; **b** Total expense (per flock) on antimicrobials by production week conditional to the presence of disease in flocks; **c** Total expense (per flock) on antimicrobials by production week regarding to flock we ekly mortality (expressed in cents of 1 US$); **d** Average cost of ADD_kg_ product by production week. Black lines in each bar represent the mean ± SE. High mortality indicates ≥2.8 birds/100 per week

The probability of disease was highest during the first week of the production cycle (0.56 SE ± 0.02), decreasing thereafter. Overall bird mortality peaked during the 5–10 week period (Fig. 2a). Of a total of 3948 weeks observed across all of 203 flocks, 1113 (28.19%) corresponded to weeks with disease (clinical signs reported) and 2835 (71.81%) to weeks without disease.

On average, farmers spent ₵125.38 (SE ± 6.76) and ₵40.0 (SE ± 1.97) on antimicrobials on their flocks in weeks with and without disease, respectively (Kruskal-Wallis *χ*^2^ = 367.3; *p* < 0.001). The (average) expense (per bird) on antimicrobials in weeks with and without disease was ₵0.34 (SE ± 0.02) and ₵0.15 (SE ± 0.01), respectively (Kruskal-Wallis χ^2^ = 315.7; *p* < 0.001). Of weeks with disease, the highest overall expense on antimicrobials corresponded to weeks 8–12, with a peak in week 10 (flock mean ₵210.81; SE ± 39.75) (Fig. 2b). Weekly mortality was categorized as low or high based on a (mean) cut-off of 2.8 per 100 birds (2.8%). The average expense on antimicrobials (per flock) in weeks with disease with high and low mortality was ₵137.86 (SE ± 8.34) and ₵157.40 (SE ± 7.98), respectively (Kruskal-Wallis χ^2^ = 2.9; *p* = 0.085) (Fig. 2c). The equivalent per bird expense was ₵0.37 (SE ± 0.02) and ₵0.47 (SE ± 0.03) for weeks with, respectively, high and with low mortality (Kruskal-Wallis χ^2^ = 1.2; *p* = 0.274). The average cost of antimicrobial products used (expressed as cost of product ADD_kg_) chosen in weeks with and without disease was, respectively, ₵0.60 (SE ± 0.04) and ₵0.54 (SE ±0.05) (Kruskal-Wallis χ^2^ = 0.4; *p* = 0.528) (Fig. 2d).

### Retail prices of antimicrobial products and AAIs

Retail prices of antimicrobial-containing products were collated from farmers’ records documenting AMU on 191 full flock cycles (raised in 100 farms). A total of 619 different health-supporting products were identified, of which 236 products contained AAIs. Data on 22 antimicrobial-containing products intended for human use (tablets) and injectable antimicrobials for animal use were excluded, since it was not clear how these were administered to flocks, the quantities used and number of birds treated. A total of 775 pricing records on the remaining 214 antimicrobial products were used to summarize retail prices. These 214 products contained 37 different AAIs belonging to 13 classes. A total of 71.9% products contained only antimicrobial active ingredients (AAIs) (apart from excipient), whilst 28.1% contained AAIs mixed with substances such as vitamins, mineral and electrolytes. Examination of the products’ labels indicated that 76 contained one AAI, 137 contained two AAIs, and one contained four AAIs (tylosin, sulphamethoxazole, sulfadiazine, and trimethoprim) (used by one farm on one flock) An additional file gives a detailed description of all 214 products (Additional file 1). Data from a total of 775 price estimates from farmers were used to summarize the price of products based on their AAI composition (see Additional file 2). A table with information on all antimicrobial products broken down by their AAIs content, the number of farms using these products and their mean retail price is given in an additional file (see Additional file 3). These are further aggregated by class of AAI in Table 1.

**Table 1:** Classification of 214 antimicrobial-containing products based on their AAI composition and WHO classification, as well as their frequency of use and retail price (based on 775 farmer pricing records)

WHO category	No. products (*N* = 214) (%)	AAIs in products (No. of products in bracket)	No. farms using (*N* = 100) (%)	No. flocks using (*N* = 191) (%)	Mean price per product ADD_kg_ (₵) (±SE)
Highest priority+High priority	43 (20.1)	colistin+amoxicillin (12), colistin+ampicillin (12), colistin+neomycin (8), colistin+gentamicin (2), colistin+apramycin (1), tylosin+gentamicin (5), tylosin+amoxicillin (2), tylosin+streptomycin (1)	65 (65.0%)	103 (53.9%)	0.52 (±0.03)
Highest priority only	41 (19.2)	enrofloxacin (12), flumequine (9), tilmicosin (6), erythromycin (2), norfloxacin (2), tylosin (1), colistin (2), colistin+tylosin (3), colistin+spiramycin (2), colistin+enrofloxacin (1), colistin+erythromycin (1)	52 (52.0%)	85 (44.5%)	0.45 (±0.07)
Highest priority +Highly important	38 (17.8)	colistin+oxytetracycline (8), colistin+doxycycline (1), colistin+lincomycin (1), colistin+sulfadimethoxine (1), doxycycline+tylosin (6), doxycyline+tilmicosin (1), erythromycin+sulphamethoxazole (2), erythromycin+oxytetracycline (1), kitasamycin+thiamphenicol (1), oxytetracycline +neomycine (2), oxytetracycline+spiramycin (3), oxytetracycline+tylosin (2), tylosin+sulfadimidine (3), tylosin+tetracycline (2), tylosin+sulfachloropyridazine (1), tylosin+sulfamethazine (1), tylosin+sulphamethoxazole (1)	88 (88.0%)	163 (85.3%)	0.36 (±0.03)
Highly important only	35 (16.4)	oxytetracycline (9), oxytetracycline+sulfadimidine (1), oxytetracycline+thiamphenicol (1), doxycycline (5), doxycycline+florfenicol (3), doxycycline+lincomycin (1), florfenicol (8), lincomycin (2), cephalexin (1), cefadroxil (1), sulphathiazole (1), sulfamethoxypyridazine+tetracycline (1), sulphamethoxazole+ thiamphenicol (1)	58 (58.0%)	77 (40.3%)	0.46 (±0.06)
High priority+Highly important	25 (11.7)	lincomycin+spectinomycin (6), doxycycline+gentamicin (5), doxycycline+neomycin (1), doxycycline+ampicillin (1), oxytetracycline+streptomycin (5), oxytetracycline+neomycin (4), ampicillin+sulfadimethoxine (1), gentamicin+sulfadimidine (1), streptomycin+sulphamethoxazole (1)	43(43.0%)	63 (33.0%)	0.45 (±0.07)
High priority only	12 (5.6)	amoxicillin (7), neomycin (3), ampicillin (1), streptomycin (1)	19 (19.0%)	23 (12.0%)	0.31 (±0.04)
Highly important +Other	8 (3.7)	sulfadimethoxine+trimethoprim (4), sulfadiazine+trimethoprim (1), sulfadimidine+trimethoprim (1), sulphamethoxazole+trimethoprim (1), doxycycline+tiamulin (1)	14 (14.0%)	14 (7.3%)	0.84 (±0.42)
Highest priority +Other	6 (2.8)	colistin+trimethoprim (3), josamycin+trimethoprim (1), spiramycin+trimethoprim (1), colistin+enramycin (1)	14 (14.0%)	14 (7.3%)	0.46 (±0.09)
Other only	3 (1.4)	trimethoprim (2), methenamine (1)	23 (23.0%)	32 (16.8%)	0.57 (±0.04)
High priority +Other	2 (0.9)	gentamicin +trimethoprim (1), neomycin+trimethoprim (1)	6 (6.0%)	6 (3.1%)	0.86 (±0.18)
Highest priority +Highly important+Other	1 (0.5)	tylosin+trimethoprim+sulfadiazine+sulphamethoxazole (1)	1 (1.0%)	1 (0.5%)	0.67 (nc)

A total of 129 (60.3%) products contained at least one critically important of the highest priority AAI; 82 (38.3%) contained at least one critically important of high priority AAI; 107 (50%) contained an antimicrobial of high importance, and 19 (8.9%) at least one antimicrobial of any other type.

**Fig. 3:**
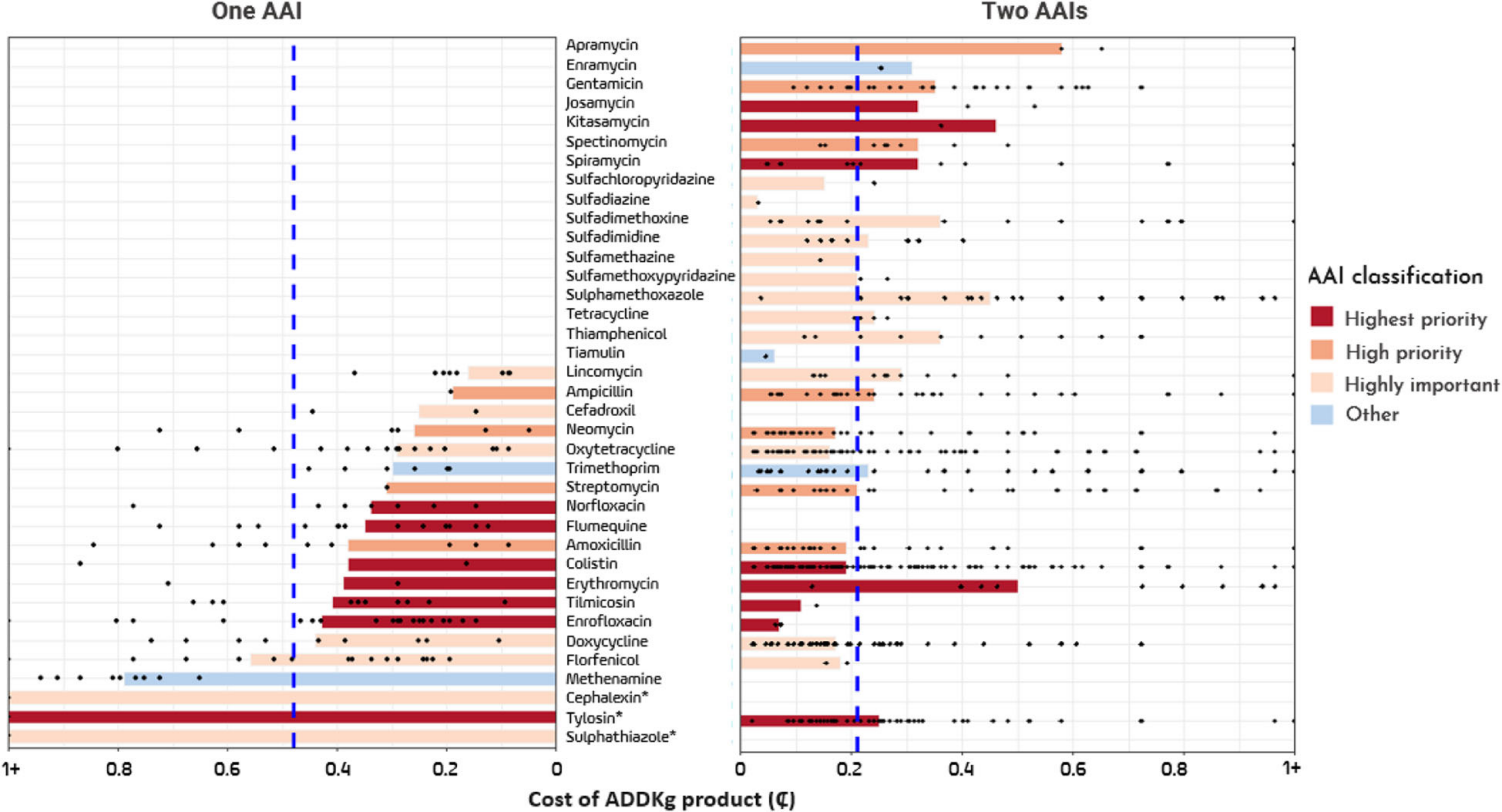
Average price per ADD_kg_ product stratified by type of AAIs contained. Separate analyses were done from products containing one or two AAIs (*cephalexin ₵2.43; sulphathiazole ₵5.43; tylosin 3.74). The blue dash line indicates the average price per ADD_kg_ (₵) across all products

The average farm-adjusted retail price of products (expressed as a daily dose of an antimicrobial product administered to a 1 kg bird, or 1 ADD_kg_) was ₵0.40 (SE ± 0.05). The retail price (per ADD_kg_ of product), from more to less affordable, corresponded to antimicrobial products containing: (1) exclusively high priority, critically important AAIs (mean ₵0.31 SE ± 0.04 per ADD_kg_); (2) highest priority, critically important in combination with highly important AAIs (₵0.36; SE ± 0.03); (3) highly important AAIs in combination with other types (mean ₵0.84; SE ± 0.42); and (4) high priority, critically important in combination with other AAIs (mean ₵0.86; SE ±0.18). With regards to products containing one AAIs, the mean retail price per ADD_kg_ ranged from ₵0.16 (SE ± 0.03) (lincomycin) to ₵5.44 (sulphathiazole) (average price ₵0.48; SE ± 0.03). With regards to products containing two AAIs, the average retail price (per ADD_kg_) corresponding to each of the AAIs contained was ₵0.21 (SE ± 0.03) ranging from ₵0.03 (SE ± nc) (sulfadiazine) to ₵0.58 (SE ±0.16) (apramycin) (Fig. 3).

### Frequency of use and price of antimicrobial products

There was no significant correlation between the frequency of use (number of weeks each product was used on flocks) and the average price of each antimicrobial product (Spearman rank *ρ* = 0.05; *p* = 0.495).

### Association between product- and farm-related factors and retail price of products

Three factors were independently associated with a higher retail price of antimicrobial-containing products: (1) Those including AAIs only (*p* = 0.007); (2) Cao Lanh district (compared with Thap Muoi) (*p* < 0.001); and (3) Type of antimicrobial. We evaluated pair-wise differences between all four categories of antimicrobials, and only differences between the high priority (the most expensive) and highly important (the least expensive) categories remained significantly different (*p* = 0.034) (Table 2).

**Table 2:** Linear random effects models investigating factors associated with the retail price of antimicrobial products. Data corresponding to 775 price measurements corresponding to 213 products containing one or two AAIs were included in the model

	Univariable			Multivariable^a^		
	*β*	SE	*p*-value	*β*	SE	*p*-value
Two AAIs (baseline = 1 AAI)	0.102	0.060	0.089			
Type of AAI included (baseline = Highly important)						
Highest priority	0.001	0.061	0.993			
High priority	0.141	0.059	0.017	0.122^b^	0.057	0.034
Other	0.111	0.101	0.274			
Pure AAI in product (baseline = mixed with other products)	0.186	0.056	< 0.001	0.152	0.056	0.007
Cao Lanh district (baseline = Thap Muoi)	0.464	0.070	< 0.001	0.468	0.071	< 0.001

^a^Intercept = −1.221 (SE = 0.062); ^b^Baseline = All other types of AAIs combined

Data corresponding to 904 weeks when AMU was reported were used to investigate farm-related factors associated with price (standardized per ADD_kg_) of antimicrobial product used. These data are shown separately (see Additional file 4). Only two factors remained significant in multi-variable model: (1) age of flock (higher ADD_kg_ retail price in older flocks) (*p* < 0.001) and (2) Cao Lanh district (*p* < 0.001) (Table 3).

**Table 3:** Linear random effects models investigating farm-related factors associated with ADD_kg_ price of antimicrobial products used. Data on 904 price estimates corresponding to weeks when farmers administered antimicrobials were used

	Univariable			Multivariable^a^		
	*β*	SE	*p*-value	*β*	SE	*p*-value
Farm owner’s age (years)(Baseline = < 36)						
36–54	0.197	0.139	0.161			
> 54	0.031	0.156	0.843			
Farm owner’s gender (Baseline = Female)						
Male	0.323	0.150	0.033			
Farm owner’s experience in raising chickens (years)(Baseline = 0–2)						
> 2–4	− 0.021	0.114	0.853			
> 4	−0.008	0.154	0.958			
Farm owners’ education attainment(Baseline = Post high school)						
Primary school	0.514	0.236	0.033			
Secondary school	0.387	0.229	0.096			
High school	0.320	0.235	0.177			
Chicken total (log)	0.061	0.061	0.319			
Age of flock (weeks) (log)	0.157	0.025	< 0.001	0.153	0.024	< 0.001
Disease status (baseline = No disease)						
Disease	0.085	0.050	0.084			
Mortality > 2.8/100 birds/week (Baseline ≤2.8/100 birds/week)	0.091	0.056	0.108			
Cao Lanh district (Baseline = Thap Muoi)	0.533	0.083	< 0.001	0.514	0.081	< 0.001

^a^Intercept = −1.331 (SE = 0.066)

## Discussion

To our knowledge, this is the first study reporting pricing of antimicrobials intended for veterinary use. Since the study is based on a large random sample of farms and a large number of products, we believe that these results accurately reflect the types of antimicrobials used and their associated prices in the Mekong Delta region of Vietnam. Much of this area shares a similar agro-ecological, demographic, as well as a similar antimicrobial retail landscape. We describe here a high diversity of AAIs used in poultry with a relatively low retail price (mean product price ₵0.40 per ADD_kg_), in line with a preliminary study [15]. Overall, retail prices did not greatly differ across WHO classes, with the exception of high priority, critically important antimicrobials, largely driven by the higher prices of apramycin, gentamicin (aminoglycosides) and spectinomycin (aminocyclitol). Over two thirds of the products (68.7%) contained two AAIs. This situation is very different in the European Union where most licenced antimicrobial products contain only one active ingredient [16].

A low retail price of antimicrobial-containing products was not reflected in a higher frequency of use. This is surprising, since often most popular consumer goods and brands tend also to be the most affordable. A study on antimicrobials of human use in Mongolia found that lower-priced antimicrobials were also those purchased more frequently e [17]. This underlines that factors other than retail price drive antimicrobial consumption intended for animal use in the Mekong Delta, and is consistent with the farmers’ perception that retail price is not a limiting factor for AMU [18]. Results indicate that the farmers are making judgements on the value of the products when confronted with disease or when treating older flocks that are more valuable. Further research in the area confirmed that farmers chose veterinary drug shops for reasons others than strict pricing, including other services such as advice, diagnostics or even loan services [19].

One of the two study districts (Cao Lanh) was associated with higher retail prices. This district is located closer to the provincial capital, and has a greater density of veterinary drug shops than the other study district. This difference confirms the existence of the variability in terms of market structure across districts. A recent study also showed a higher frequency of AMU in farms located in this district [20], suggesting that demand for these products could be partly responsible for higher prices. This observation, however, does raise the question of whether marginal changes in price will reduce AMU in small-scale livestock systems.

Regardless of the AAI contained, antimicrobial products that contained pure AAIs were more expensive than AAIs mixed with other substances. These products were generally imported and likely to be more expensive. Details on the compounding and wholesale of AAIs requires further investigation.

Notably, in terms of frequency and expressed per kg of live animal, AMU was highest during the first week of the life of flocks. However, the greatest overall expense corresponded to the 8–12 week period, where the incidence of mortality was highest and birds had reached a higher bodyweight. This is consistent with a previous study when mortality was highest during the same period [20]. The older age of the flock was also associated with the choice of more expensive antimicrobial products. These results indicate that farmers are sensitive to the antimicrobial products’ cost in relation to their perceived potential effectiveness. Therefore, farmers use more expensive antimicrobials in the face of disease threat, as well as to protect the higher value of older birds. A previous study indicated that giving antimicrobials to chicken flocks made farmers feel more secure, since more expensive antimicrobials are also perceived to be more effective [18].

The study provides conclusive evidence that prices of antimicrobial-containing products used in chicken production systems in the Mekong Delta are extremely low. Even though many of these products contained AAIs of high importance to human medicine, this is not reflected in the retail price. Retail price is only a component of cost for accessing antimicrobials, the other relates to travel to retail sites and barriers restricting their purchase. In the study area, access to retail points is relatively easy, with farmers located on average ~ 2 km from their closest veterinary drug shop [19]; once there the purchase involves a simple request for the product of preference or a consultation describing the flock health and the needs. There is currently no need for prescription (i.e. veterinary fee), therefore the retail cost is an accurate reflection of the actual cost to the farmer of using antimicrobials in their livestock production system.

It has been stated that overuse of antimicrobials and antimicrobial resistance is partly the result of a dysfunctional health system, and that antimicrobial stewardship requires long-term commitment to healthcare provision [21]. Policies need to strike a balance between access to antimicrobials by those that really need them whilst preventing unnecessary use [22]. A potential measure that may reduce excessive AMU includes the compulsory requirement of a prescription for purchasing antimicrobials intended for veterinary use similar to that currently in place in some developed countries [23]. However, given the large size of the farming community and the limitations of the veterinary services in Vietnam, such policy would be difficult to implement in the short- to mid-term. Levying a tax on antimicrobial products intended for animal use has been suggested as a policy intervention with a potential to reduce excessive AMU [24]. In view of these results, we consider that levying taxes on the most critical important antimicrobial categories would be reasonable policy intervention.

## Conclusions

Our study provides conclusive evidence of the comparatively low prices of antimicrobial-containing products used in chicken production systems in the Mekong Delta of Vietnam, and their lack of relatedness with their human medicine relevance. Implementing pricing mechanisms that provide a signal to retailers and farmers and that the products they are selling and using (antimicrobials) are of importance to society is a policy measure worth exploring. We recommend that retail surveys of antimicrobials should be conducted in other areas within Vietnam as well as other countries in region, so that large-scale pricing policy interventions may be implemented. Any changes in pricing policies would require careful monitoring of the demand response of retailers and farmers whilst ensuring lack of adverse effect on animal health. Such work would provide a true basis for evidence-based policy on the pricing of antimicrobial-containing veterinary products.

## Materials and methods

### Study flocks and data collection

The study was conducted in Dong Thap province (Mekong Delta). Farmers were randomly chosen from the census (2015) of chicken farmers of two districts (Cao Lanh and Thap Muoi). All flocks investigated corresponded to the baseline (i.e. observational) phase of an intervention study. The aim was to recruit 120 farms raising 100–2000 chickens per cycle [25]. There were 13,264 and 5371 registered chicken farms in Cao Lanh and Thap Muoi districts. According to this census, 275 (Cao Lanh) and 201 (Thap Muoi) farms had a capacity of 100–2000 chickens. A total of 207 farmers raising > 100 chickens according to the 2015 census were randomly chosen and were contacted by letter by the veterinary authorities (sub-Department of Animal Health and Production of Dong Thap, SDAHP). A meeting was held with 199 attending farmers (96%), in which the project aims and methods were introduced. Farmers were asked to contact the project team should they wish to restock within the following 6 months. A total of 102 (51.3%) such farmers restocking with 100–2000 chickens contacted the project team within 6 months of the meeting and expressed their willingness of being enrolled in the study. Each participating farmer was given a purposefully designed diary alongside a large plastic container. Farmers were asked to weekly record in the diary information on the number of chickens, presence of disease and the amounts of any health-related products used, their costs, and the route of administration (oral-water, oral-feed, injectable) in the diaries. They were also asked to keep all containers of any health-related product in the plastic container, as well as the receipts reflecting the purchases of these products. A research team visited each farm on four different times during the duration of each flock production cycle (typically 3–5 months). On the day of the visit, information on the antimicrobial products recorded in the diaries was compiled, and pictures were taken of the products’ labels. The data were subsequently uploaded onto a central database. The pictures of the labels of all health-related products administered to the flocks were carefully examined to determine which products contained AAIs, their strength and the mode of administration. Recruited farms were investigated from October 2016 to March 2018.

### Data analyses

Retail prices paid by farmers to purchase antimicrobial products for oral administration were compiled. The retail prices of each product was standardized to ‘amount required to treat one kg of live chicken’ (ADD_kg_). This was calculated based on the manufacturers’ guidelines on product preparation for therapeutic purposes (dilution of the product in water and/or feed), the retail costs of the product (from farmer’s diaries), and the estimated ‘daily intake’ of a 1 kg chicken (estimated in 225 ml water or 63 g feed). For products with an indication for both water and feed preparation, indications for dilution in water were followed. Prices were expressed in cents of 1 US$ (₵), based on an exchange rate of 1 US$ = 23,319 VND (as of 23rd September 2018).

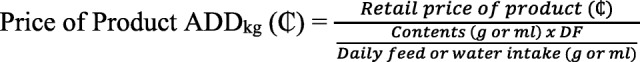

$$ {\displaystyle \begin{array}{l}\mathrm{DF}=\mathrm{Dilution}\ \mathrm{factor}\ \Big(\mathrm{volume}\ \mathrm{or}\ \mathrm{weigh}\mathrm{t}\ \mathrm{of}\ \mathrm{antimicrobial}\ \\ {}\mathrm{product}\ \mathrm{related}\ \mathrm{to}\ \mathrm{volume}\ \mathrm{or}\ \mathrm{weigh}\ \mathrm{of}\ \mathrm{water}\ \mathrm{or}\ \mathrm{feed}\left)\right)\end{array}} $$

The probability of AMU by week was calculated by dividing the number of flocks using antimicrobials by the number of flocks observed across all weeks. The total expense on antimicrobial products over the production cycle was calculated by week from usage data, and was related to the number and weight of birds, as well as the presence/absence of disease in the flock. The weight of birds in flocks by week was estimated from a previous study [1]. We compared the farmers’ expense on antimicrobials in weeks with and without disease, stratified by level of flock mortality (computed by the number of dead birds divided by the total birds at the beginning of that week). The average retail price of chosen antimicrobial products (expressed in ADD_kg_) was computed stratified by flock age and disease status. Comparisons between retail prices of antimicrobials used at different ages and between flocks with and without disease were performed using the Kruskal-Wallis statistic.

The correlation between frequency of use and the average price of each antimicrobial product (standardized as ADD_kg_) was investigated using the Spearman rank correlation coefficient. The frequency of use was expressed as the number of weeks using of each antimicrobial containing products was used.

The price associated with each specific AAI contained in antimicrobial products was expressed in relation to 1 kg of chicken treated with the product (ADD_kg_). These were calculated by dividing the price of the product by 1, 2 or 4, depending on the number of AAIs included.

Antimicrobial products were then classified by their AAI composition according to the WHO criteria: (1) ‘Highest priority, critical important’, (2) ‘High priority, critical important’, (3) ‘Highly important’, and (4) ‘Other’.

The potential association between product-related factors and their retail price to the farmer (expressed as ADD_kg_ product) was investigated by building a random effect multivariable linear model with ‘Farm’ specified as a random effect. Factors investigated as fixed-effect covariates were: (1) Number of AAIs in the product (one or two); (2) Type of AAIs based on WHO classification; (3) Product composition (‘only AAIs’ or ‘AAIs mixed with other substances’ in the product); and (4) farm district location (Thap Muoi, Cao Lanh). We also investigated the association between farm- and farmer-related factors and retail price by building an additional model with he following covariates as fixed effects: (1) Farm owner’s age (log); (2) Farm owner’s gender; (3) Farm owner’s experience in poultry farming (years); (4) Farm owner’s highest education attainment; (5) Flock size (number of chickens) (log); (6) Age the flock (weeks) (log); (7) Flock disease status (yes/no); (8) Flock weekly mortality; (9) District location (Cao Lanh/Thap Muoi). A step-wise forward approach was followed in model building. First univariable models were built, and variables with an associated *p* < 0.20 were screened for multivariable analyses. Only variables with *p* ≤ 0.05 were retained in the final multivariable model. Final model residuals were checked for normality and outliers were excluded in a subsequent analysis. All analyses were carried out using R software (version 3.4.3) with the ‘lme4’ and ‘lmerTEST’ packages.

## Supplementary information

**Additional file 1.** Description of 214 antimicrobial-containing products. Description of each of 214 antimicrobial-containing products, their AAI content, as well as their price.

**Additional file 2.** Price estimates of each of antimicrobial-containing products. Price paid by farmers for each purchase of a single antimicrobial-containing product.

**Additional file 3.** Table summarizing retail costs of one Animal Daily Dose administered to 1 kg chicken (ADDkg) for 213 products containing one or two AAI each.

**Additional file 4.** Average cost of antimicrobials (ADDkg) used by week in farms. Average cost of ADDkg of antimicrobials given by farms in relation to flock-related variables.

## Data Availability

The datasets used in the manuscript are available as additional files.

## Abbreviations

AAI: Active antimicrobial ingredient
ADD: Animal daily dose
AMU: Antimicrobial usage
CIAs: Critically important antimicrobials
DF: Dilution factor
IQR: Inter-quartile range
LMICs: Low- and middle-income countries
SDAH-DP: Sub-Department of Animal Health and Production of Dong Thap
SE: Standard error
ViParc: Vietnamese Platform for Antimicrobial Reductions in Chicken production
WHO: World Health Organization
