# Supplementary material for: A survey of retail prices of antimicrobial products used in small-scale chicken farms in the Mekong Delta of Vietnam
Source: Global Health. 2020 Jan 14;16:8. doi: 10.1186/s12992-019-0539-x (PMC6961362; doi:10.1186/s12992-019-0539-x)
Supplement: Supplementary file 3 — Additional file 3. Table summarizing retail costs of one Animal Daily Dose administered to 1 kg chicken (ADDkg) for 213 products containing one or two AAI each. [file 12992_2019_539_MOESM3_ESM.docx]

| Antimicrobial classes | AAI | Products with 1AAI (n=74) | | |  | Products with 2AAIs (n=139) | | |  | All products (n=213) | |
| --- | --- | --- | --- | --- | --- | --- | --- | --- | --- | --- | --- |
|  |  | No. of products | No. of purchases | Mean retail price  (±SE)  ADDkg (₵) |  | No. of products | No. of purchases | Mean retail price  (± SE)  ADDkg (₵) |  | No. of purchases | Mean retail price  (± SE)  ADDkg (₵) |
| Macrolides^**^ | Erythromycin | 2 | 4 | 0.39 (±0.14) |  | 4 | 23 | 0.50 (±0.06) |  | 27 | 0.49 (±0.06) |
|  | Kitasamycin | - | - | - |  | 1 | 1 | 0.46 (±nc) |  | 1 | 0.41 (±nc) |
|  | Josamycin | - | - | - |  | 1 | 4 | 0.32 (±0.06) |  | 4 | 0.36(±0;06) |
|  | Tilmicosin | 6 | 36 | 0.41 (±0.04) |  | 1 | 1 | 0.11 (±nc) |  | 37 | 0.36 (±0.04) |
|  | Spiramycin | - | - | - |  | 6 | 15 | 0.32 (±0.09) |  | 15 | 0.33 (±0.09) |
|  | Tylosin | 1 | 1 | 3.74(±nc) |  | 28 | 163 | 0.25 (±0.02) |  | 167 | 0.07(±0.04) |
| Polymyxins^**^ | Colistin | 2 | 2 | 0.38 (±0.35) |  | 59 | 459 | 0.19 (±0.01) |  | 461 | 0.19(±0.01) |
| Quinolones^**^ | Enrofloxacin | 12 | 73 | 0.43 (±0.15) |  | 1 | 3 | 0.07 |  | 76 | 0.40 (±0.14) |
|  | Flumequine | 9 | 28 | 0.35 (±0.04) |  | - | - | - |  | 28 | 0.35 (±0.04) |
|  | Norfloxacin | 2 | 13 | 0.34 (±0.06) |  | - | - | - |  | 13 | 0.34 (±0.06) |
| Aminoglycosides^*^ | Apramycin | - | - | - |  | 1 | 3 | 0.58 (±0.16) |  | 3 | 0.61(±0.16) |
|  | Gentamicin | - | - | - |  | 14 | 84 | 0.35 (±0.02) |  | 84 | 0.34(±0.02) |
|  | Spectinomycin | - | - | - |  | 6 | 13 | 0.32 (±0.15) |  | 13 | 0.31(±0.15) |
|  | Streptomycin | 1 | 2 | 0.31 (±nc) |  | 7 | 77 | 0.21(±0.04) |  | 79 | 0.21(±0.04) |
|  | Neomycin | 3 | 21 | 0.26 (±0.07) |  | 14 | 69 | 0.17 (±0.04) |  | 90 | 0.19 (±0.03) |
| Penicillins^*^ | Ampicillin | 1 | 1 | 0.19 (±nc) |  | 14 | 74 | 0.24 (±0.04) |  | 75 | 0.25(±0.04) |
|  | Amoxicillin | 7 | 19 | 0.38 (±0.06) |  | 14 | 69 | 0.19 (±0.03) |  | 88 | 0.22(±0.03) |
| 1^st^ & 2^nd^ gen. cephalosporins | Cephalexin | 1 | 1 | 2.43 (±nc) |  | - | - | - |  | 1 | 2.43 (±nc) |
|  | Cefadroxil | 1 | 2 | 0.25 (±0.15) |  |  | - | - |  | 2 | 0.25 (±0.15) |
| Lincosamides | Lincomycin | 2 | 9 | 0.16 (±0.03) |  | 8 | 16 | 0.29 (±0.12) |  | 25 | 0.24(±0.08) |
| Amphenicols | Florfenicol | 8 | 35 | 0.56 (±0.11) |  | 3 | 4 | 0.18 (±0.01) |  | 39 | 0.46(±0.1) |
|  | Thiamphenicol | - | - | - |  | 3 | 36 | 0.36 (±0.03) |  | 36 | 0.36(±0.03) |
| Sulfonamides | Sulphathiazole | 1 | 1 | 5.44(±nc) |  | - | - | - |  | 1 | 5.44 (±nc) |
|  | Sulphamethoxazole | - | - | - |  | 6 | 68 | 0.45 (±0.03) |  | 71 | 0.45(±0.03) |
|  | Sulfadimethoxine | - | - | - |  | 6 | 21 | 0.36 (±0.19) |  | 21 | 0.36(±0.19) |
|  | Sulfamethoxypyridazine | - | - | - |  | 1 | 4 | 0.21 (±0.02) |  | 4 | 0.21(±0.02) |
|  | Sulfadimidine | - | - | - |  | 6 | 11 | 0.23 (±0.03) |  | 11 | 0.21(±0.03) |
|  | Sulfamethazine | - | - | - |  | 1 | 1 | 0.21 (±nc) |  | 1 | 0.21 (±nc) |
|  | Sulfachloropyridazine | - | - | - |  | 1 | 1 | 0.15 (±nc) |  | 1 | 0.15(±nc) |
|  | Sulfadiazine | - | - | - |  | 1 | 1 | 0.03 (±nc) |  | 4 | 0.07(±nc) |
| Tetracyclines | Tetracycline |  | - | - |  | 3 | 7 | 0.24 (±0.01) |  | 7 | 0.24 (±0.01) |
|  | Doxycycline | 5 | 28 | 0.44 (±0.05) |  | 20 | 103 | 0.17 (±0.02) |  | 131 | 0.2 (±0.01) |
|  | Oxytetracycline | 9 | 41 | 0.29 (±0.05) |  | 28 | 318 | 0.16 (±0.01) |  | 359 | 0.17(±0.01) |
| Polypeptides | Enramycin | - | - | - |  | 1 | 1 | 0.31 (±nc) |  | 1 | 0.31 (±nc) |
| Pleuromutilins | Tiamulin | - | - | - |  | 1 | 1 | 0.06 (±nc) |  | 1 | 0.06 (±nc) |
| Diaminopyrimidines | Trimethoprim | 2 | 17 | 0.30 (±0.04) |  | 14 | 53 | 0.23 (±0.09) |  | 73 | 0.24(±0.07) |
| Unclassified class | Methenamine | 1 | 31 | 0.79 (±0.02) |  | - | - | - |  | 31 | 0.79(±0.02) |

^**^Critically important, highest priority; ^*^Critically important, high priority; nc=Not calculated.
